# Regenerative Therapies for Equine Degenerative Joint Disease: A Preliminary Study

**DOI:** 10.1371/journal.pone.0085917

**Published:** 2014-01-20

**Authors:** Sarah Broeckx, Marieke Zimmerman, Sara Crocetti, Marc Suls, Tom Mariën, Stephen J. Ferguson, Koen Chiers, Luc Duchateau, Alfredo Franco-Obregón, Karin Wuertz, Jan H. Spaas

**Affiliations:** 1 Global Stem cell Technology, Meldert-Lummen, Belgium; 2 Equine Veterinary Practice Dr Suls, Weert, The Netherlands; 3 Department for Biomechanics, ETH Zurich, Zurich, Switzerland; 4 Equitom Equine Hospital, Meldert-Lummen, Belgium; 5 Competence Center for Applied Biotechnology and Molecular Medicine CABMM, University of Zurich, Zurich, Switzerland; 6 Department of Pathology, Bacteriology and Poultry Diseases, Faculty of Veterinary Medicine, Ghent University, Merelbeke, Belgium; 7 Department of Comparative Physiology and Biometrics, Faculty of Veterinary Medicine, Ghent University, Merelbeke, Belgium; 8 Department of Surgery, Yong Loo Lin School of Medicine, National University of Singapore, Singapore; Instituto Butantan, Brazil

## Abstract

Degenerative joint disease (DJD) is a major cause of reduced athletic function and retirement in equine performers. For this reason, regenerative therapies for DJD have gained increasing interest. Platelet-rich plasma (PRP) and mesenchymal stem cells (MSCs) were isolated from a 6-year-old donor horse. MSCs were either used in their native state or after chondrogenic induction. In an initial study, 20 horses with naturally occurring DJD in the fetlock joint were divided in 4 groups and injected with the following: **1**) PRP; **2**) MSCs; **3**) MSCs and PRP; or **4**) chondrogenic induced MSCs and PRP. The horses were then evaluated by means of a clinical scoring system after 6 weeks (T_1_), 12 weeks (T_2_), 6 months (T_3_) and 12 months (T_4_) post injection. In a second study, 30 horses with the same medical background were randomly assigned to one of the two combination therapies and evaluated at T_1_. The protein expression profile of native MSCs was found to be negative for major histocompatibility (MHC) II and p63, low in MHC I and positive for Ki67, collagen type II (Col II) and Vimentin. Chondrogenic induction resulted in increased mRNA expression of aggrecan, Col II and cartilage oligomeric matrix protein (COMP) as well as in increased protein expression of p63 and glycosaminoglycan, but in decreased protein expression of Ki67. The combined use of PRP and MSCs significantly improved the functionality and sustainability of damaged joints from 6 weeks until 12 months after treatment, compared to PRP treatment alone. The highest short-term clinical evolution scores were obtained with chondrogenic induced MSCs and PRP. This study reports successful *in vitro* chondrogenic induction of equine MSCs. *In vivo* application of (induced) MSCs together with PRP in horses suffering from DJD in the fetlock joint resulted in a significant clinical improvement until 12 months after treatment.

## Introduction

Degenerative joint disease (DJD) is a major cause of reduced athletic function and retirement in equine performers [1]–[3]. Medical treatment for DJD may include anti-inflammatory and analgesic drugs to reduce inflammation and pain, and so-called disease-modifying drugs such as glucosamine, chondroitin sulphate or hyaluronic acid [4]–[6]. In the case of severe cartilage and bone degeneration, the use of articular cartilage curettage, osteophyte removal or even arthrodesis could be suitable [4], [7]. Nevertheless, the aforementioned therapies are merely aimed at alleviating the symptoms or enhancing clinical recovery, without inducing an actual regeneration of the affected joint.

The field of equine regenerative medicine is drawing increasing attention in the scientific community for its treatment strategies of joint pathologies. Equine mesenchymal stem cells (MSCs) are of specific therapeutic interest as they can differentiate *in vitro* towards cells with a hyaline-like cartilage morphology and produce cartilage-specific components such as collagen type II and glycosaminoglycans [8]–[10]. Moreover, horses may serve as a valuable large animal model for the evaluation of new human therapies concerning *in vivo* efficiency and safety, due to interspecies similarities in tendon structure [11], [12] as well as thickness of the non-calcified cartilage of the stifle joint [13]. Therefore, the evaluation of new treatments for musculoskeletal injuries in horses may be of broad clinical benefit for both equine and human medicine.

Other than a single case report with a positive clinical outcome of naturally occurring DJD after MSC therapy [14], the few available placebo-controlled studies in horses consist of experimentally induced cartilage lesions [15]–[17], not entirely resembling the clinically observed pathology. Importantly, the micro-environment - or niche - in degenerated cartilage might not provide the correct signals for MSC differentiation or alternatively, may even negatively influence their viability. Therefore, a priori chondrogenic induction of MSCs may improve the clinical outcome. In fact, this approach of provoking tenogenic induction has been used in the past to treat different equine tendon lesions, with promising clinical results [18], [19]. Therefore, a principal aim of this study was to evaluate the clinical effects of a combined therapy for the treatment of equine DJD, using either native MSCs plus platelet-rich plasma (PRP) or chondrogenic induced MSCs plus PRP. This approach was compared to the more conventional regenerative therapies based on the use of PRP or native MSCs alone, which have been shown to be clinically safe [18]–[26]. This study also sought to compare the therapeutic efficacies of chondrogenically-induced MSCs (plus PRP) to native MSCs (plus PRP).

Allogenic peripheral blood (PB) from one donor horse was used as the source of MSCs, since it has been previously reported that PB MSCs also have the capacity to produce cartilage *in vitro* [10], [27]. Moreover, the same donor horse could be used to produce PRP, thus substantially increasing the standardization of the sample production and the comparability between the different treatment groups: **1**) PRP alone (n = 5); **2**) native MSCs alone (n = 5); **3**) native MSCs and PRP (n = 5); and **4**) chondrogenic induced MSCs and PRP (n = 5). Chondrogenic induction was assessed *in vitro* by immunocytochemistry and real-time RT-PCR analysis. A clinical scoring system was established in order to enable two non-blinded independent veterinarians to give their professional assessment of the clinical status of the injured joint. At different time points after treatment (6 weeks, 12 weeks, 6 months and 12 months), scores were given to all the patients.

## Materials and Methods

This study was carried out in strict accordance with the recommendations of the Animal Welfare Department of the Belgian Federal Public Service of Health. The protocol was approved by the Committee on the Ethics of Animal Experiments of Global Stem cell Technology (Permit Number: LA1700607). All injections were performed after sedating the horses, and all efforts were made to minimize suffering.

### Isolation and Chondrogenic Induction of Mesenchymal Stem Cells (MSCs)

In total, 50 ml of blood was collected in sterile EDTA tubes from the *vena jugularis* of a 6-year-old donor gelding, which was tested for different transmittable diseases at Böse laboratory (Harsum, Germany), as previously reported by our group [19]. Approval of the ethical committee was obtained (EC_2012_001). In order to isolate mesenchymal stem cells (MSCs), the blood sample was centrifuged at 1000 G for 20 minutes and the buffy coat was collected and diluted 1∶2 in phosphate buffered saline (PBS) 1x. Afterwards, this suspension was gently layered on an equal amount of Percoll® density gradient (GE Healthcare). The further isolation and characterization was performed as previously described [10].

After that, 20×10^6^ peripheral blood mononuclear cells (PBMCs) were seeded per T_75_ flask in 3 flasks and expanded in culture medium consisting of low glucose (LG) DMEM, 20% foetal calf serum (FCS) and 1% antibiotics-antimycotics (AB/AM) [10]. The medium was refreshed twice a week and the cells were maintained at 37°C and 5% CO_2_. At 60% confluency, the cells were trypsinized with 0.25% trypsin-EDTA and subcultured until passage 3, at which time cells were characterized as previously described [10] before seeding them at 6.7×10^3^ MSCs/cm^2^ in T_75_ flasks for expansion, or chondrogenic induction. Chondrogenic induction medium consisted of DMEM LG, 20% FCS, 1% AB/AM and cartilage-specific growth factors, similar to a previous report by Jonitz [28]. At the next confluency, native and chondrogenic induced cells were trypsinized, resuspended in 1 ml of DMEM LG with 10% of dimethyl sulfoxide (DMSO, Sigma) and frozen before being shipped on dry-ice for clinical application (Arti-Cell® and Arti-Cell® Plus respectively).

### Preparation of Platelet-rich Plasma (PRP)

In total, 300 ml of peripheral blood was taken in a citrate phosphate dextrose adenine-1 (CPDA-1) single blood bag (Terumo®) for platelet-rich plasma (PRP) preparation. From this donor horse, 30 samples of 1 ml PRP were prepared as previously described by our group [18], [19]. Each sample contained approximately 200×10^6^ platelets and was frozen and stored at −80°C before clinical application.

### Cytological Staining

Hematoxylin (HE), Crystal Violet (CV), Alcian Blue (AB) and Safranin O (SO) staining (all from Sigma) were performed on MSCs and chondrogenic-induced MSCs, as indicated by the manufacturer. Both HE and CV staining were carried out, in order to visualize the cell morphology and cellular organization. Furthermore, AB and SO staining were performed to give an indication of the presence of acid polysaccharides, such as glycosaminoglycans in cartilage-like structures.

### Immunocytochemistry

Immunocytochemistry was performed to evaluate the expression of collagen type II (Col II), Ki67 (proliferation marker), p63 (tumor suppression gene) and vimentin (mesenchymal cell marker) on native MSCs and chondrogenic-induced MSCs in adhesive tissue culture plates and after trypsinization and cytospin preparation at 700 rpm for 4 minutes. Cells were fixed for 10 minutes with 4% PF and permeabilized for 2 minutes with 0.1% Triton X at room temperature. Subsequently, cells were incubated with hydrogen peroxide (0.03%) for 5 minutes at room temperature and after washing with PBS, incubated for 30 minutes at room temperature with the primary rabbit IgG polyclonal antibodies recognizing: Col IIA1 (1∶50), Ki67 (1∶200) and p63 (1∶100) and mouse IgG_1_ monoclonal anti-vimentin (1∶100) (all from Abcam). After washing with PBS, secondary ready-to-use goat anti-mouse and anti-rabbit peroxidase (PO)-linked antibodies (Dako) were added and incubated for 30 minutes at room temperature. Finally, 3,3′-diaminobenzidine (DAB) was added for 5 minutes and a counter staining with hematoxylin was performed to visualize the surrounding cells. As controls, identical staining was performed on undifferentiated MSCs and background staining was assessed by using the proper isotype-specific mouse monoclonal or rabbit polyclonal antibody. All isotypes were matched to the immunoglobulin subtype and used at the same protein concentration as the corresponding antibodies. Wherever appropriate, equine tendon or skin tissue sections were used as negative controls.

### Flow Cytometry

To characterize the MSCs immunophenotypically, the expression of several stem cell markers was evaluated by flow cytometry, as previously described [10]. For the present study, we evaluated the expression of the typical rejection proteins, major histocompatibility (MHC) class I and II on native and chondrogenic induced MSCs. Per series, 400’000 cells were used and labeled with the following primary antibodies: mouse anti-horse MHC class I IgG_2a_ (Washington State University, 1∶50) and mouse anti-horse MHC class II IgG_1_ (Abd Serotec, 1∶50). Cells were incubated with the primary antibodies for 15 minutes on ice in the dark and washed twice in washing buffer, consisting of DMEM with 1% bovine serum albumin (BSA). A secondary rabbit anti-mouse-FITC (Abcam, 1∶100) antibody was used to identify positive cells after 15 minutes of incubation on ice in the dark. Finally, all cells were washed three times in washing buffer and at least 10′000 cells were evaluated using a fluorescence activated cell sorter (FACS). All analyses were based on (i) autofluorescence and (ii) control cells incubated with isotype-specific IgG’s, in order to establish the background signal. All isotypes were matched to the immunoglobulin subtype and used at the same protein concentration as the corresponding antibodies. As positive controls, PBMCs were used to confirm MHC cross-reactivity.

### Gene Expression Analysis by Real-time RT-PCR

Equine MSCs in passage 3 were seeded in T_25_ flasks at a density of 8′000 MSCs/cm^2^ with expansion medium or chondrogenic induction medium for 30 hours. After treatment, cells were lysed in 2 ml of Trizol (Invitrogen) and the lysate was separated into aqueous and organic phases by chloroform separation (300 µl, Sigma-Aldrich). The aqueous phase was recovered after centrifugation and total RNA was precipitated by using equal volumes of isopropanol. The precipitate was washed with 75% EtOH once and then solubilized with 25 µl of RNAse free water and quantified on the Nanodrop Lite (Fisher Scientific) before reverse transcribing 1 µg of RNA, using the TaqMan Reverse Transcription Reagents Kit (Life Technologies). Gene expression analysis was performed in triplicate (30 ng of cDNA in each reaction) with TaqMan Gene Expression Assays (Life Technologies) (Table 1) on the CFX96 Real-Time PCR System (Biorad). Values were normalized to GAPDH mRNA as internal control and presented as fold change, compared to native MSCs (i.e. in expansion medium), using the comparative CT method ( = 2−ΔΔCT method).

**Table 1:** TaqMan gene expression assays used for real-time RT-PCR.

Target gene	Assay ID
Aggrecan	Ec03469667_m1
Collagen II	Ec03467386_g1
COMP	Ec03468079_g1
GAPDH	Ec03210916_gH

### Patient Inclusion Criteria

For a first study, 20 acceptor horses were selected based on their injuries. To be included in this study, clinical lameness had to be present in a mild to moderate form for at least 3 months. Moreover, the observed locomotory disorder had to be attributable to fetlock (metacarpophalangeal or metatarsophalangeal) joint osteoarthritis. In this regard, the source of the lameness was confirmed by both local analgesia and a positive flexion test for all the patients. In all included horses, the lameness was exacerbated by a flexion test of the fetlock joint, and was abolished by intra-articular administration of a local anaesthetic solution. In the present study, 5 ml of 0.5% Mepivacaine Hydrochloride (Meaverin Actavis®) solution was used, and horses were evaluated 10 minutes after injection. Furthermore, for horses to be included, radiographic (X-ray) or computer-tomographic (CT) signs of osteoarthritis of the fetlock joint had to be noticeable in the form of osteophytes and/or cartilage defects. For a second study (comparing 2 combination treatments), 30 horses were selected using the same inclusion criteria. Untreated or placebo animals could not be included in the present study, since only owner horses with naturally occurring DJD were used.

### Injecting Mesenchymal Stem Cells (MSCs) and Monitoring of Adverse Reactions

For each horse, the intra-articular injection was performed at least 24 hours after local anaesthesia, since it has been reported that exposure of MSCs to high concentrations of anaesthetics negatively influences cell viability [29]. In addition, 0.04 mg/kg detomidine (Domosedan®) and 0.1 mg/ml butorphanol (Turbogesic®) were administered intravenously, for their sedative and analgesic effects, respectively. In the first study, horses were randomly assigned to PRP, native MSCs, native MSCs and PRP (Combination 1), or chondrogenic-induced MSCs and PRP (Combination 2) treatment. In the second study, horses were randomly assigned to one of the two combination therapies. After thawing, both MSCs and PRP were aspirated in the same syringe (for combination groups) and administered intra-articulary. After the treatment, the horses were closely monitored for 1 week by means of a daily examination of the injected joint and by observing the occurrence of possible adverse effects or hypersensitivity reactions (wheal formation, sweating, strong respirations or even fever). Subsequently, the joints were evaluated at approximately 6 weeks (T_1_), 12 weeks (T_2_), 6 months (T_3_) and 12 months (T_4_) post injection through clinical evaluation by 2 independent veterinarians for all horses. In the second study, horses were randomly assigned to one of the two combination therapies and evaluated at T_1_. The ethical committee approved the experimental design (EC_2013_001).

### Clinical Scoring System

In order to evaluate the severity of the clinical condition, the following parameters were graded by the same veterinarians at the aforementioned time points (T_0–4_): clinical lameness from 0 to 5 (0 =  no lameness and 5 =  minimal weight bearing lameness) according to the American Association of Equine Practitioners (AAEP), response to flexion test from 0 to 3 (0 =  no flexion response and 3 =  severe flexion response) and fetlock joint effusion from 0 to 2 (0 =  no swelling and 2 =  severe swelling). Because the importance of each parameter was correlated with its impact, the sum of these 3 parameters was reckoned as the overall clinical severity score (0 to 10), with 0 corresponding to clinical soundness. A detailed overview of the different scores can be found in Table 2.

**Table 2:** All the patients were clinically assessed for joint effusion, response to flexion test and lameness according to the American Association of Equine Practitioners (AAEP).

	Score	Clinical implication
**Joint** **effusion**	0	No swelling
	1	Moderate swelling
	2	Severe swelling
**Flexion** **test**	0	No flexion response
	1	Mild flexion response
	2	Moderate flexion response
	3	Severe flexion response
**AAEP** **grading**	0	No lameness
	1	Lameness not consistently regardless circumstances
	2	Lameness consistently under certain circumstances
	3	Lameness consistently observable on a straight line
	4	Obvious lameness: marked nodding or shortened stride
	5	Minimal weight bearing lameness in motion or at rest

Because the importance of each parameter was correlated with its impact, the sum of these 3 parameters was reckoned as the overall clinical severity score (0 to 10).

All the horses in this study showed initially a mild to moderate lameness (1–2 out of 5), mild to moderate response to flexion test (1–2 out of 3) and moderate to severe joint effusion (1–2 out of 2). As a result, all horses had a very similar initial clinical score of 4–5 out of 10. Progress was scored relative to before the treatment. Since none of the patients worsened, all the scores were greater than zero and translated in a positive evolution score ranging from 0 to 5∶0 =  severity score of 5 out of 10; 1 =  severity score of 4 out of 10; 2 =  severity score of 3 out of 10; 3 =  severity score of 2 out of 10; 4 =  severity score of 1 out of 10; and 5 =  return to clinical soundness or severity score of 0 out of 10. Severity scores were translated to evolution scores for easier interpretation of the data and a positive trend would therefore indicate a clinical improvement. Statistical analysis was performed based upon the clinical evolution scores.

### Statistical Analysis

For data analysis in study 1, the average of the evolution scores at 6 and 12 weeks represented the early evolution score, and the average of the evolution scores at 6 and 12 months represented the late evolution score. The early and late evolution scores are compared between the group receiving both MSCs (either native or induced) and PRP and the group receiving only MSCs on the one hand or receiving only PRP on the other hand, using the Wilcoxon signed rank sum test at the 5% significance level. Furthermore, within the combined treatment (study 2), the chondrogenic-induced MSCs are compared with native MSCs only for the earliest evolution score (i.e. at 6 weeks) equally using the Wilcoxon signed rank sum test at the 5% significance level.

## Results

### Isolation of Mesenchymal Stem Cells (MSCs)

The first spindle shaped cells were noticed after 17 days in culture and were isolated at 21 days at approximately 60% confluency. The characterization experiments revealed the same MSC properties as previously described [10], with the addition of several markers.

### Characterization and Chondrogenic Induction of MSCs

To initially characterize MSCs and confirm chondrogenic induction, we analyzed cell morphology by light microscopy utilizing Hematoxylin and Crystal Violet staining. Biochemical induction was analyzed by measuring gene and protein expression of selected cell markers (glycosaminoglycan production, collagen type II (Col II), Ki67 p63, vimentin, major histocompatibility complex, aggrecan, cartilage oligomeric matrix protein) providing insight into the degree of chondrogenic-induction by real-time RT-PCR, Alcian Blue staining, Safranin O staining, immunocytochemistry and flow cytometry.

**Figure 1:**
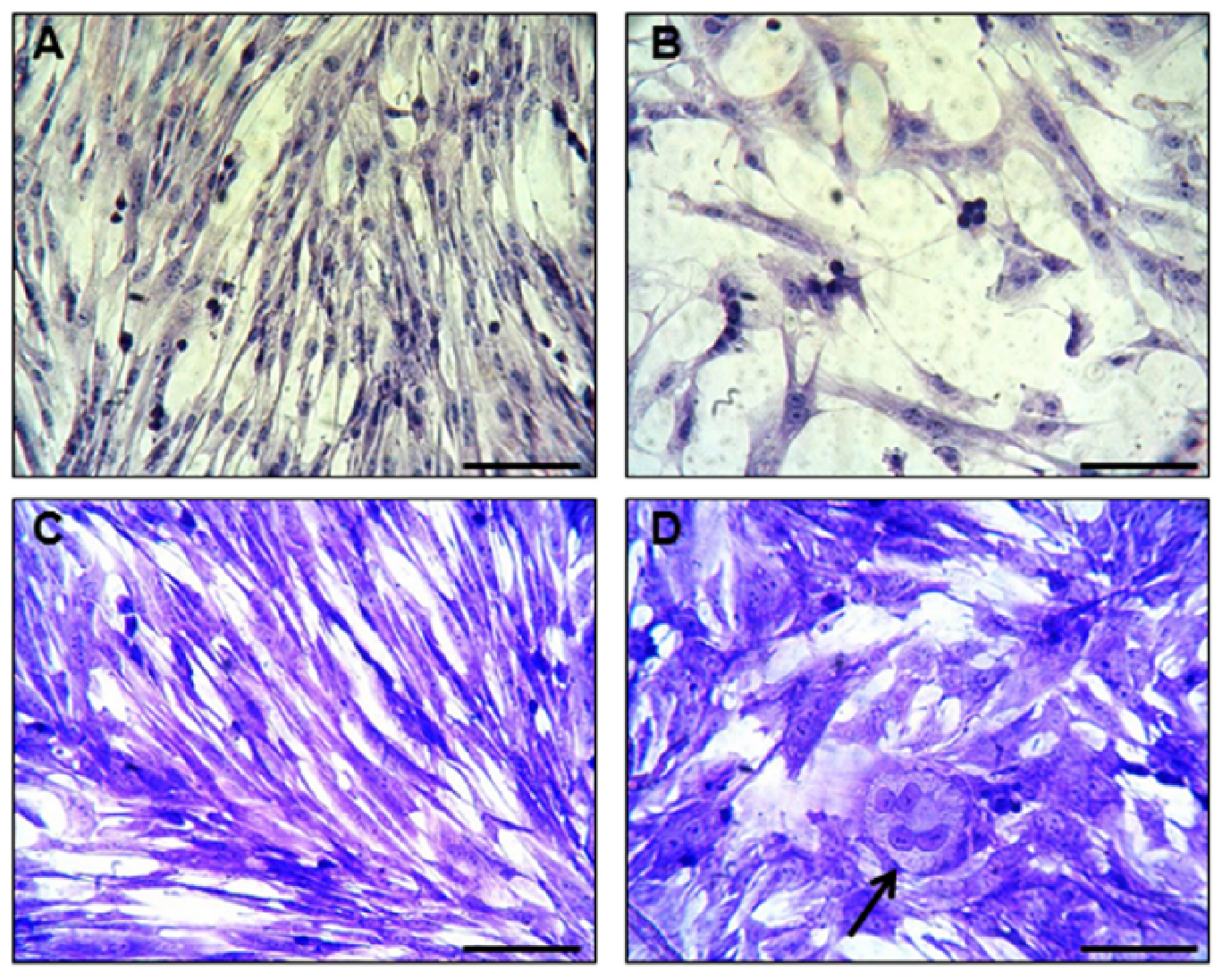
Representative images of peripheral blood (PB)-derived mesenchymal stem cells (MSCs) in their undifferentiated state (A & C) and chondrogenic induced (B & D) after Hematoxylin (A & B) and Crystal Violet (C & D) stainings. The typical chondrogenic morphology and lacune formation (black arrow) can be noticed after induction. Scale bars represent 50 µm.

**Figure 2:**
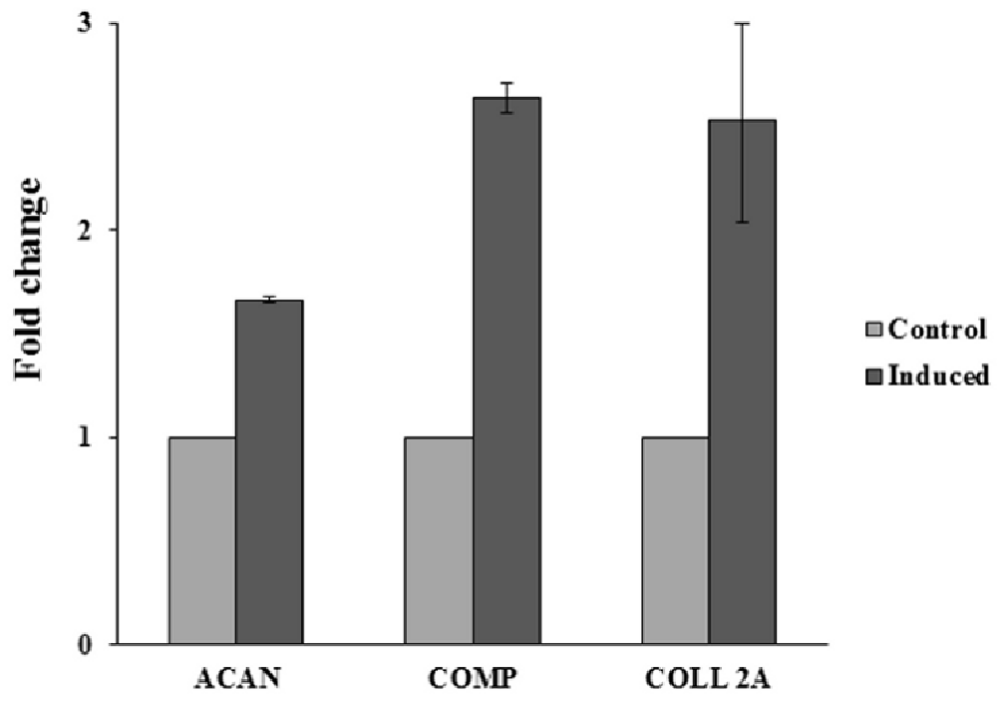
Results of RT-PCR for the gene expression of collagen (Col) type II, aggrecan and cartilage oligomeric matrix protein (COMP) in the native MSCs (Ctrl) and chondrogenic induced MSCs (Ind). Values are given as the mean of three measurements ± SEM.

**Figure 3:**
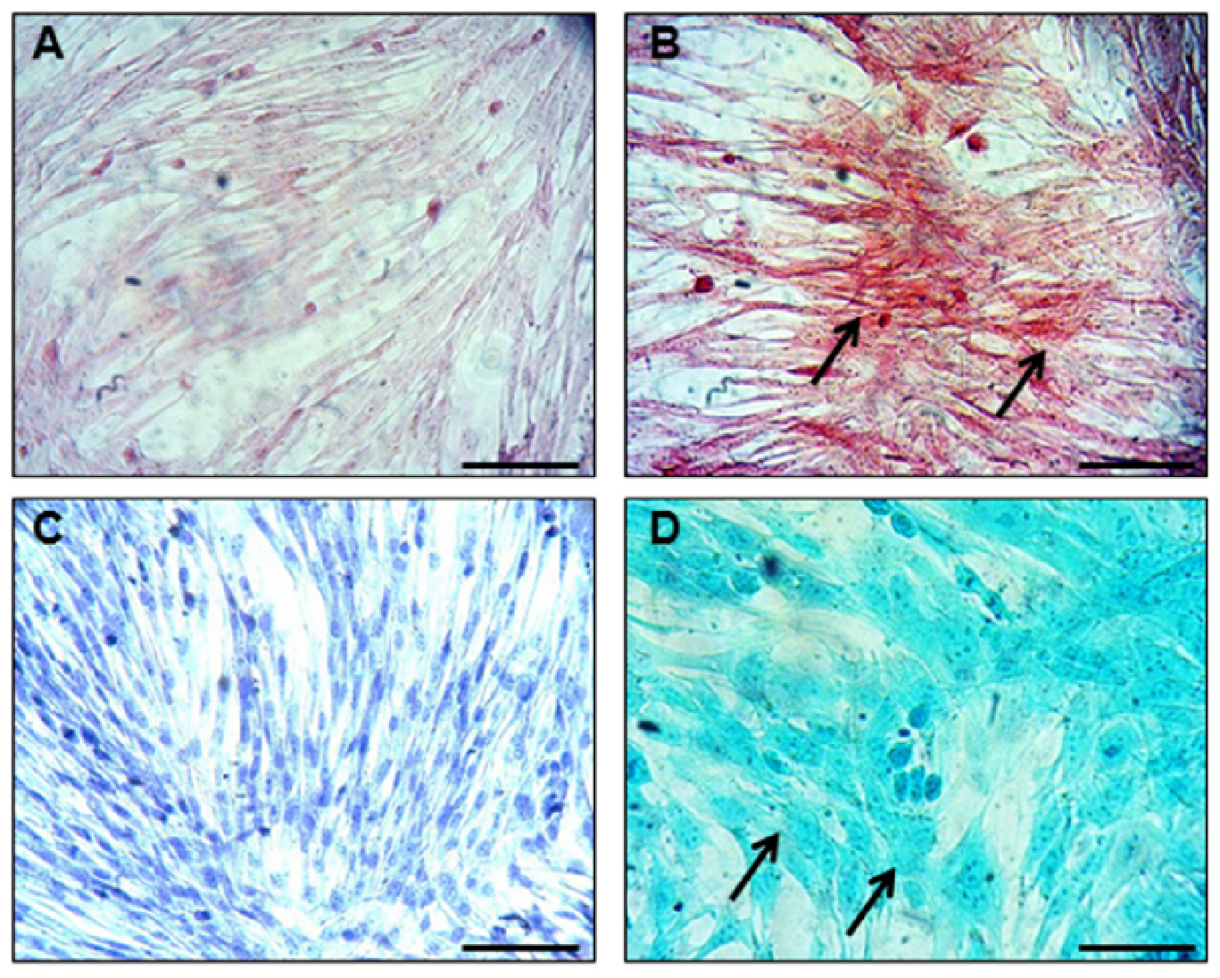
Representative images of peripheral blood (PB)-derived mesenchymal stem cells (MSCs) in their undifferentiated state (A & C) and chondrogenic induced (B & D) after Alcian Blue (A & B) and Safranin O (C & D) stainings. Glycosaminoglycan production (black arrows) can be noticed after induction. Scale bars represent 50 µm.

Light microscopic analysis in conjunction with HE and Crystal Violet staining showed that native MSCs (Figure 1A & C) had a stellate/spindle-shaped morphology and displayed a propensity to grow in colonies, whereas MSCs induced into the chondrogenic lineage (Figure 1B & D) showed a more rectangular morphology. In addition, a few chondrocyte-like cells in lacune-like structures could be noticed after 3 days of culturing in the chondrogenic-inducing medium (Figure 1D). Gene expression analysis confirmed the switch towards a chondrogenic phenotype, exhibiting increases in the levels of Col II, aggrecan (ACAN) and cartilage oligomeric matrix protein (COMP) in induced MSCs, compared to native MSCs (Figure 2). Histological staining of the cells with both Alcian Blue and Safranin O confirmed the production of glycosaminoglycans in the chondrogenic-induced group (Figure 3B & D), whereas undifferentiated MSCs stained negative (Figure 3A & C).

**Figure 4:**
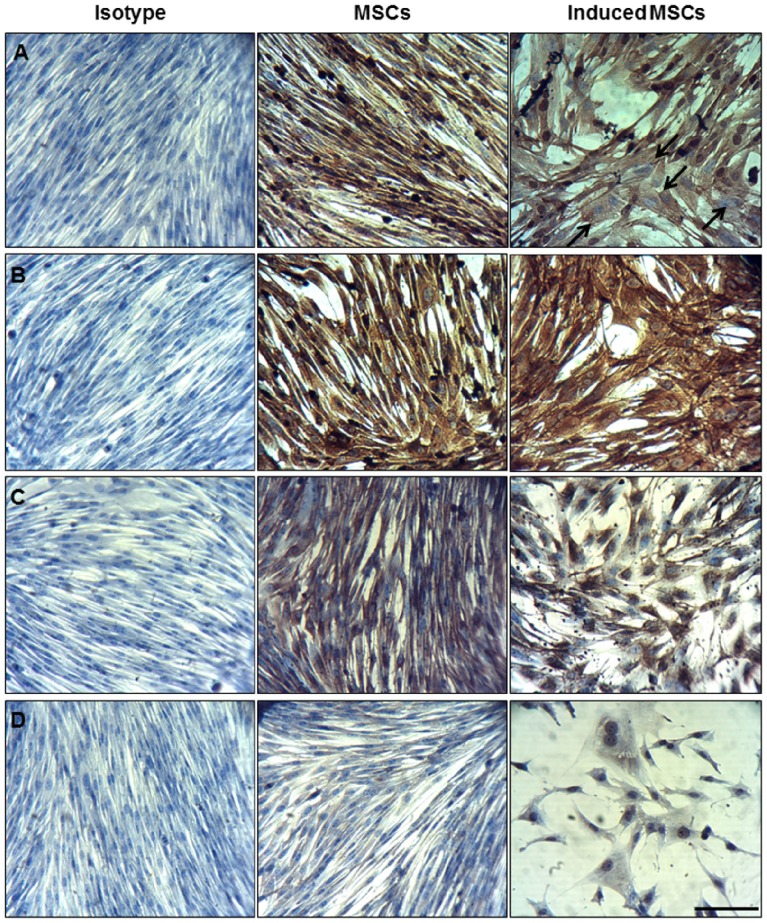
Immunocytochemistry on adhesive mesenchymal stem cells (MSCs) using Ki67 (A), collagen (Col) type II (B), vimentin (C) and p63 (D). Native MSCs were negative for p63 and positive for Ki67, Col II and vimentin, whereas chondrogenic induced MSCs were positive for p63, Col II and vimentin with a decreased signal for Ki67 (arrows = negative nuclei). The relevant isotype controls were negative. Scale bar represents 50 µm.

Immunocytochemistry in adhesion (Figure 4) as well as after trypsinization and cytospin preparation (Figure S1) revealed that most of the nuclei in the native MSC group were positive for the proliferation marker Ki67, whereas noticeably less nuclei stained positively in the chondrogenic-induced group (Figure 4A, Figure S1A). Moreover, native MSCs and chondrogenic-induced MSCs were both positive for Col II (Figure 4B, Figure S1B). Adhesive culture and cytospin analysis further indicated that native and chondrogenic-induced MSCs were immunoreactive for vimentin (Figure 4C, Figure S1C), while p63 (Figure 4D, Figure S1D), which is a member of the p53 tumor suppressor gene family, was only detectable in chondrogenic-induced MSCs. Isotype (Figure 4, Figure S1) and negative controls (data not shown) stained negative.

**Figure 5:**
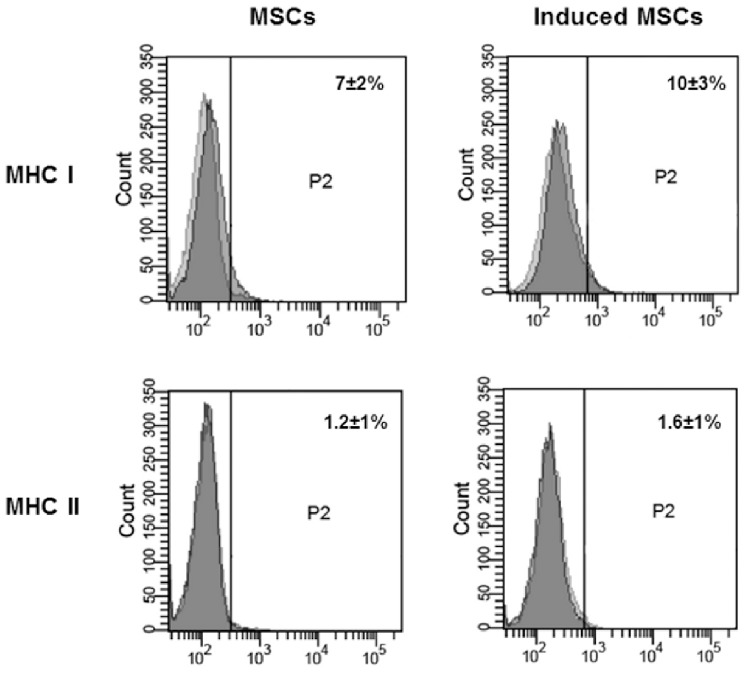
Flow cytometry confirmed a low expression of major histocompatibility complex (MHC) class I and no expression of MHC class II on the native MSCs and chondrogenic induced MSCs. The light and dark grey histograms represent the relevant isotype control staining and marker antibody staining, respectively with the corresponding percentage of mean positive cells ± SEM.

*In vitro* differentiation towards undesired lineages (i.e. myogenic, endothelial, or smooth muscle differentiation) results in an increase of the expression of typical rejection proteins, major histocompatibility complex (MHC) classes I and II [30]. It is thus of relevance that our differentiation protocol showed no increase in these markers. While MHC class II expression was completely absent in both native and chondrogenic-induced MSCs, MHC class I was expressed in both types of MSCs, but at very low levels (Figure 5A & B). The positive control cells (peripheral blood mononuclear cells) on the other hand, were clearly positive for both MHC markers and confirmed antibody cross-reactivity (data not shown).

### Scoring of the Clinical Lameness

#### Study 1

**Figure 6:**
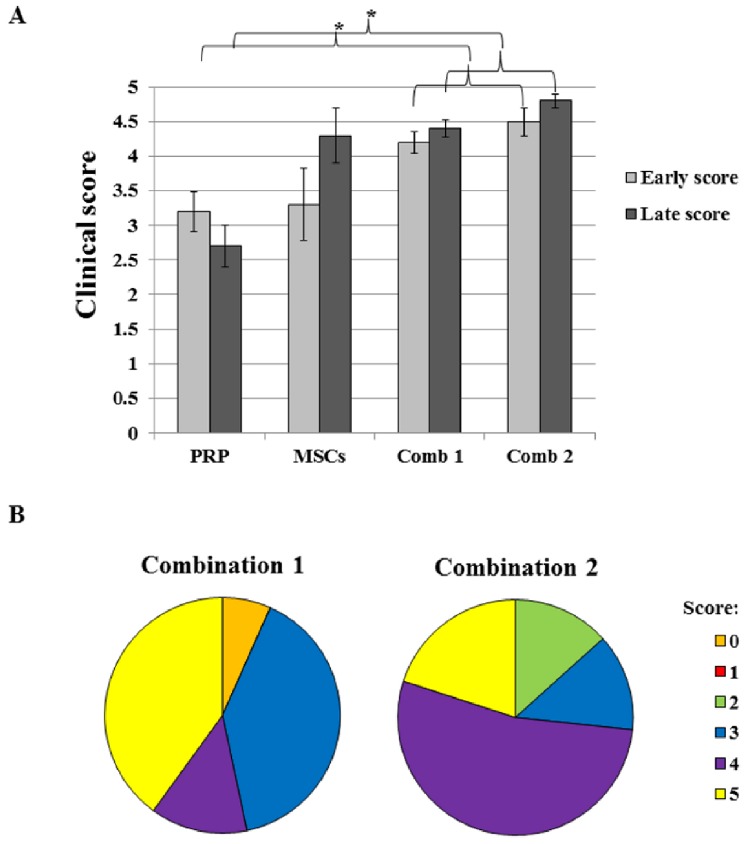
Clinical evolution scores of the different treatments at different time points in the first study (A). Values are given as the mean ± SEM. Diagrams represent the clinical evolution scores of 30 horses treated with native mesenchymal stem cells (MSCs) and PRP (Combination 1, n = 15) or chondrogenic induced MSCs and PRP (Combination 2, n = 15) in the second study (**B**).

The grading and overall clinical severity scores with the corresponding initial severity scores can be found in the Figure S2. All the patients enrolled in this study had similar clinical scores immediately prior to the onset of treatment and for each patient the clinical evolution scores were calculated at the termination of the experiment. The scores for each treatment group were hence clinically comparable (Table 3). The platelet-rich plasma (PRP) treated group (#1) initially (6 weeks post injection) received an average score of 3.4, which is higher than for the MSC treated group (#2). By combining both PRP and MSCs (group #3) the initial average score was increased. The clinical score improved further in the chondrogenic induced MSCs and PRP combinational therapy (group #4). Subsequently, the average score of the PRP treated group (#1) decreased to 2.6 at one year after the treatment, indicating that the effect was short-lived. The initial average score of 3.0 in group #2 was the lowest for all the treatment groups, due to one non-responder. The average score for group #2, however, increased to 4.4 at 6 months, and then decreased to 4.2 at one year after the treatment. Horses in group #3 had a higher initial average score of 3.8, analogous to the PRP treated group, that increased to an average score of 4.2 at one year post injection, likely attributed to the long-term effects of the MSCs. Noteworthy, the average score of group #4 was initially 4.4 and increased further to 4.8 from 6 months to one year post injection. Moreover, two horses in group #4 showed functional recovery as early as 6 weeks after commencing treatment and remained sound throughout the entire study. Indeed, all horses in group #4 exhibited at least a score 4 at 6 weeks after treatment; 4 out of 5 horses in group #4 were sound at one year post injection and one horse had a score of 4 throughout the entire study period. For the first study, an overview of the average evolution scores after each treatment is presented in Figure 6A. To get a stronger short-term (early) versus long-term (late) clinical evolution in response to the different therapies the scores at 6 weeks and 12 weeks and 6 months and 12 months were added together (Table 4). The combined treatments were significantly better than the PRP treatment alone, both for the early evolution score (P = 0.033) and the late evolution score (P = 0.012). No significant differences were found between the combined treatment and the MSC treatment alone. The combined use of chondrogenic-induced MSCs and PRP generated the highest evolution scores, although the difference was not significantly higher than the combined use of native MSCs and PRP for either the early (P = 0.530) or late evolution score (P = 0.207).

**Table 3:** Clinical evolution scores with average and standard deviation (STD) at different time points for the different treatment groups: 1 = platelet-rich plasma (PRP), 2 = mesenchymal stem cells (MSCs), 3 = PRP+MSCs, and 4 = PRP+chondrogenic induced MSCs.

		6 weeks	12 weeks	6 months	12 months
**GROUP 1**	Horse 1	4	2	2	1
	Horse 2	4	5	4	4
	Horse 3	3	3	3	3
	Horse 4	3	2	2	2
	Horse 5	3	3	3	3
	**Average**	**3.4**	**3**	**2.8**	**2.6**
	STD	0.5	1.2	0.8	1.1
**GROUP 2**	Horse 6	3	4	5	5
	Horse 7	0	1	2	2
	Horse 8	3	4	5	5
	Horse 9	4	4	5	4
	Horse 10	5	5	5	5
	**Average**	**3**	**3.6**	**4.4**	**4.2**
	STD	1.9	1.5	1.3	1.3
**GROUP 3**	Horse 11	3	4	4	5
	Horse 12	4	5	5	3
	Horse 13	4	5	5	4
	Horse 14	5	5	5	5
	Horse 15	3	4	4	4
	**Average**	**3.8**	**4.6**	**4.6**	**4.2**
	STD	0.8	0.5	0.5	0.8
**GROUP 4**	Horse 16	4	4	5	5
	Horse 17	5	5	5	5
	Horse 18	4	4	4	4
	Horse 19	5	5	5	5
	Horse 20	4	5	5	5
	**Average**	**4.4**	**4.6**	**4.8**	**4.8**
	STD	0.5	0.5	0.4	0.4

**Table 4:** Median, minimum (min) and maximum (max) of the early and late evolution score are indicated per treatment: platelet-rich plasma (PRP), native mesenchymal stem cells (MSCs), combination (Comb) 1 (native MSCs and PRP) or Comb 2 (chondrogenic induced MSCs and PRP).

Treatment	Early score median (min; max)	Late score median (min; max)
**PRP**	3.0 (2.5; 4.5)	3.0 (1.5; 4.0)
**MSCs**	3.5 (0.5; 5.0)	5.0 (2.0; 5.0)
**Comb 1**	4.5 (3.5; 5.0)	4.5 (4.0; 5.0)
**Comb 2**	4.5 (4.0; 5.0)	5.0 (4.0; 5.0)

The “early” score indicates the average of the clinical evolution scores at 6 weeks and 12 weeks, whereas “late” indicates the average of the clinical evolution scores at 6 months and 12 months after treatment.

#### Study 2

For this reason, the second clinical study was performed in which a total of 30 horses were treated with either native MSCs plus PRP (Combination 1, n = 15) or with chondrogenic-induced MSCs plus PRP (Combination 2, n = 15). The horses were only evaluated at the first time point (i.e. 6 weeks post injection). Our results show that 53% (8/15) of the horses in the first group received an evolution score 4 or more, versus 73% (11/15) in the second group. However, in both treatment groups the average evolution score was approximately the same (3.7 vs 3.8) and no statistically significant (P = 0.67) difference could be noticed (Table 5, Figure 6B).

**Table 5:** Clinical evolution scores at 6 weeks after treatment of 15 horses with native mesenchymal stem cells (MSCs) and PRP (Combination 1) or chondrogenic induced MSCs and PRP (Combination 2).

	Combination 1	Combination 2
**Horse 1**	3	3
**Horse 2**	5	4
**Horse 3**	4	4
**Horse 4**	4	4
**Horse 5**	3	3
**Horse 6**	5	4
**Horse 7**	5	4
**Horse 8**	3	5
**Horse 9**	5	4
**Horse 10**	3	5
**Horse 11**	3	4
**Horse 12**	0	2
**Horse 13**	5	5
**Horse 14**	5	4
**Horse 15**	3	2
**Average**	3.7	3.8

## Discussion

The isolated cells in the present study fulfilled all the requirements to be typed as mesenchymal stem cells (MSCs) according to the proposed guidelines by Dominici in 2006 [31]. Moreover, it has been reported that frozen equine peripheral blood (PB)-derived MSCs do not lose their stem cell characteristics [32] and that fresh equine PB-derived MSCs dramatically decline in cell number after 12 hours of transport and have a higher risk of becoming senescence after 24 hours of transport [33]. Therefore, the use of frozen samples was justified in this study, added to the product shelf life and standardized the treatments.

This study reports successful *in vitro* chondrogenic induction of equine PB-derived MSCs, followed by an *in vivo* investigation in which the therapeutic potential of chondrogenic-induced MSCs plus platelet-rich plasma (PRP) for the treatment of degenerative joint disease (DJD) was compared to native MSCs and/or PRP in 20 horses and both combination therapies in 30 horses. *In vitro* analysis of chondrogenic-induced MSCs showed decreased expression of the proliferation marker Ki67, indicating terminal differentiation with reduced proliferative capacities of the MSCs. Chondrogenic differentiation was further confirmed by increased mRNA levels of aggrecan, collagen type II and cartilage oligomeric matrix protein (COMP) as well as increased synthesis of glycosaminoglycans.

Apart from these typical chondrogenic markers, we also investigated the expression of the typical “rejection” proteins, major histocompatibility complex (MHC) class I and II, as well as of p63, a member of the p53 tumor suppressor gene family. MHC expression was evaluated because it has been reported that differentiation of allogenic MSCs towards myogenic lineages induced immunogenicity (by increasing MHC levels) [30]. In the present study, we were able to demonstrate that MHC class I was expressed at low levels in native MSCs, whereas neither MHC class II nor p63 were expressed in native MSCs, in agreement with previous reports [10], [34]–[36]. Three days of chondrogenic induction did not alter MHC levels, reflecting low immunogenicity, which would be permissive for allogenic transplantations. However, chondrogenic induced cells clearly expressed p63, which is a typical epithelial stem cell marker [37], [38]. In this regard, it has been reported that p63 plays a pivotal role in embryonic skeletal development and that p63 expression in hypertrophic chondrocytes would accelerate endochondral ossification [39]. Whether the expression of this protein enhanced cartilage repair in this study remains to be shown.

In the present study, we applied PRP and MSCs either alone, or in combination (with or without chondrogenic induction). Usage of PRP was anticipated to improve the clinical outcome as it has been reported that PRP enhances MSC proliferation and chondrogenic differentiation [40]. Although no statistically significant improvement in clinical signs of fetlock joint arthrosis could be noticed after the addition of PRP to MSCs (in comparison to MSCs alone), short-term clinical evolution scores clearly improved (3 vs 3.8 at 6 weeks and 3.6 vs 4.6 at 12 weeks post injection). Moreover, both combination therapies significantly improved the early and late clinical evolution scores in comparison to PRP treatment alone. The effect presented here differs from that of a previous study in deep digital flexor tendon lesions in Bergamasca sheep, where the addition of autologous PRP to PB-derived MSCs did not enhance regeneration [41]. In rat Achilles tendon lesions on the other hand, synergistic effects of PRP and tendon stem cells were demonstrated and resulted in the increased expression of tendon-healing genes [42]. In agreement with our study, it has also been reported that short-term beneficial effects might be expected from PRP treatment, although the exact mechanism or causative agent(s) are currently unknown [43]. It should also be taken into consideration that patient to patient heterogeneity at the time of blood sampling would result in PRP samples of varied potencies, between independent donors and within a given donor, helping account for the contradictory results obtained in previous studies [44]. Clearly, more research is warranted to determine the stimulatory or inhibitory factors present in PRP samples.

As we utilized only one batch of allogenic PRP and MSCs in the present study (i.e. PRP and MSCs from one donor), the experimental paradigm was more standardized for all patients, allowing a more accurate comparison between the different treatment groups. In this regard, Carrade et al. have reported that a single intra-articular injection of allogenic MSCs in healthy equine joints induced a similar immune response as an autologous injection [22]. Moreover, in a study by Guest et al., no cell-mediated immune response was detected at all after allogenic MSC injection in equine superficial digital flexor tendon lesions [45]. Analogously, in this study, there were no indications of an immune response after allogenic MSC or PRP treatment. Nonetheless, no definite conclusion can yet be made concerning the immunogenicity of both allogenic therapies used in this study.

The major advancement of this study is the application of MSCs in equine patients with naturally occurring fetlock joint arthrosis, rather than in horses with experimentally induced cartilage lesions. The fact that experimentally induced cartilage lesions may only partially resemble the naturally occurring arthrosis may explain why previous studies [15]–[17] were not able to detect clinical improvement, in contrast to our study. However, while clinical improvement was absent in the aforementioned studies, an early beneficial impact on histologic appearance and biochemical composition [17] as well as a late enhancement of aggrecan levels [16] was observed. Frisbie et al. [15] reported no significant clinical or histological effect within 70 days after treatment with bone marrow-derived MSCs in the middle carpal joint of horses, but did observe improvements in synovial fluid PgE2 levels, which would ultimately inhibit the production of pro-inflammatory cytokines [46].

In contrast with previous equine reports, and in agreement with the present study, it has been described that carpal joint arthrosis in donkeys improved clinically and radiographically at 2 months and 6 months after treatment with bone marrow-derived MSCs [47]. Moreover, green fluorescent protein-labelled MSCs integrated in the cartilage, which indicated that the MSCs participated in the healing process of the damaged tissue. Whether the difference in location and structural composition of the joints, the experimental model, the MSC samples, or even the carrier used in the aforementioned studies were responsible for the lack of clinical improvement remains to be proven.

While our study provides evidence of clinical improvement with MSC therapy in the fetlock joint, it must be noted that the initial clinical severity scores were mild to moderate (4–5 on 10), indicating that these patients were not in the last stage of osteoarthritis. Clearly, the mechanisms underlying this effect are unclear and will need to be investigated in the future. Furthermore, patients in earlier and later stages of osteoarthritis and larger sample numbers under double-blinded evaluation criteria will eventually need to undergo a similar procedure as described here to improve statistical power and allow for more definite conclusions. In our first preliminary study reported here, only five horses per treatment were evaluated, which could have been the reason why no statistical significant difference was observed between the evolution scores of both combination therapies (i.e. PRP+native MSCs versus PRP+induced MSCs). Although in a larger group of patients substantially more horses received a score of 4 or more in the second combination therapy, the average evolution scores of both combination therapies were not significantly different. Indeed, further optimization is necessary to ultimately assess both therapies.

In conclusion, our results indicate that chondrogenic induction can be achieved in equine MSCs and that the combined use of PRP and MSCs (chondrogenic induced or not) significantly improved the functionality and sustainability of damaged joints in horses with mild to moderate lameness, due to fetlock joint osteoarthritis, up to 12 months post treatment. The highest clinical scores were noticed upon treatment with the chondrogenic induced MSCs and PRP. Nonetheless, more protracted studies need to be performed to confirm the positive effects of chondrogenic induction.

## Supporting Information

Figure S1 **Immunocytochemistry on cytospins using Ki67 (A), collagen (Col) type II (B), vimentin (C) and p63 (D). Native mesenchymal stem cells (MSCs) were negative for p63 and positive for Ki67, Col II and vimentin, whereas chondrogenic induced MSCs were positive for p63, Col II and vimentin and slightly positive for Ki67.** Arrows indicate a decreased signal for Ki67 in some chondrogenic induced MSCs. The relevant isotype controls were negative. Scale bar represents 25 µm.
(TIF)

Figure S2 **Clinical grading and severity scores for the different treatment (PRP = platelet-rich plasma, MSC = mesenchymal stem cell, IND = chondrogenic induced mesenchymal stem cell) groups at different time points.** The sum of the clinical grading according to the American Association of Equine Practitioners (AAEP) on 5, the flexion test on 3 and joint effusion evaluation on 2 gave an overall clinical severity score on 10.
(TIF)
