# Supplementary figures and images for: Regenerative Therapies for Equine Degenerative Joint Disease: A Preliminary Study
Source: PLoS One. 2014 Jan 20;9(1):e85917. doi: 10.1371/journal.pone.0085917 (PMC3896436; doi:10.1371/journal.pone.0085917)

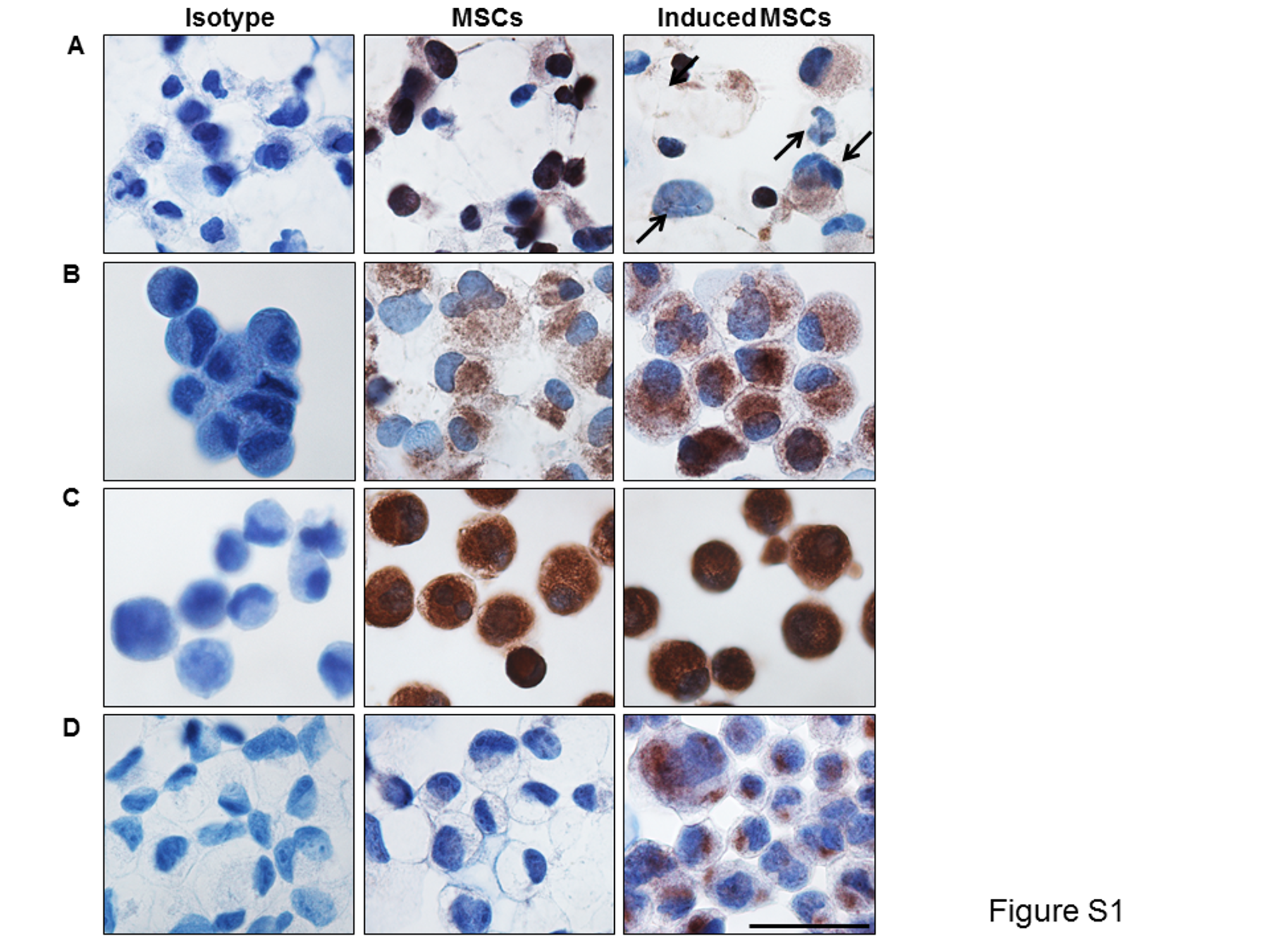

Supplement: Figure S1 — Immunocytochemistry on cytospins using Ki67 (A), collagen (Col) type II (B), vimentin (C) and p63 (D). Native mesenchymal stem cells (MSCs) were negative for p63 and positive for Ki67, Col II and vimentin, whereas chondrogenic induced MSCs were positive for p63, Col II and vimentin and slightly positive for Ki67. Arrows indicate a decreased signal for Ki67 in some chondrogenic induced MSCs. The relevant isotype controls were negative. Scale bar represents 25 µm. (TIF) [file pone.0085917.s001.tif]
